# QTL mapping and transcriptomic analysis of fruit length in cucumber

**DOI:** 10.3389/fpls.2023.1208675

**Published:** 2023-08-21

**Authors:** Yanan Xing, Yilin Cao, Yanan Ma, Fu Wang, Shijie Xin, Wenying Zhu

**Affiliations:** ^1^ Qingdao Agricultural University, Qingdao, China; ^2^ Engineering Laboratory of Genetic Improvement of Horticultural Crops of Shandong Province, Qingdao, China; ^3^ Yantai Yeda Investment Development Group Co., Ltd, Yantai, China

**Keywords:** cucumber, fruit length, QTL mapping, transcriptomic analysis, plant hormone

## Abstract

A total of 151 recombinant inbred lines (RILs) were derived from the cross between ‘*Cucumis sativus* L. *hardwickii*’ (HW) and a cultivated Northern Chinese inbred line ‘XinTaiMiCi’ (XTMC). We used resequencing to construct the genetic map and analyze the genetic background of RIL population, and combined with the phenotypes of RIL population and the analysis of RNA-seq data, we located the major loci controlling the fruit length of cucumber and related analysis. A genetic map containing 600 bin markers was constructed via re-sequencing. Based on the phenotype data collected in two different seasons (spring 2021 and autumn 2022), the major quantitative trait loci (QTLs) controlling cucumber fruit length were located and their transcriptomic analysis carried out. The results revealed three QTLs (*Fl2.1*, *Fl4.1*, and *Fl6.1*) detected repeatedly in the two seasons, of which *Fl4.1* was the dominant QTL. From the functional annotation of corresponding genes there, we discovered the gene *Csa4G337340* encoding an auxin efflux carrier family protein. The expression of that gene was significantly lower in XTMC and the long-fruit RIL lines than in HW and the short-fruit RIL lines; hence, we speculated the gene could be negatively correlated with the fruit length of cucumber. Transcriptomic analysis showed that 259 differentially expressed genes (DEGs) were enriched in the plant hormone signal transduction pathway. In addition, among those DEGs, 509 transcription factors were detected, these distributed in several transcription factor gene families, such as bHLH, AP2/ErF -ERF, C2H2, and NAC. Therefore, we concluded that the major gene controlling the fruit length of cucumber is located in the interval of *Fl4.1*, whose gene *Csa4G337340* may be involved in the negative regulation of fruit length. Further, genes related to plant hormone signal transduction and several transcription factors were also found involved in the regulation of cucumber fruit length. Our results provide a reference for the fine mapping of major genes and analyzing the mechanism of cucumber fruit length.

## Introduction

The fruit of cucumber (*Cucumis sativus*) is an important reproductive organ, whose quality of appearance and flavor directly affect whether consumers want to buy and eat it. In particular, the length of fruit is a critical agronomic trait that affects the yield and appearance quality, serving also as the main reference standard for the classification of fresh commercial fruit products (Qi et al., 2013). The fruit length of wild cucumber plants is only about 3-5 cm, whereas that of cultivated cucumber has changed significantly after its long-term domestication and improvement. Accordingly, fruit length is also a valuable trait targeted in current cucumber breeding efforts.

**Figure 1:**
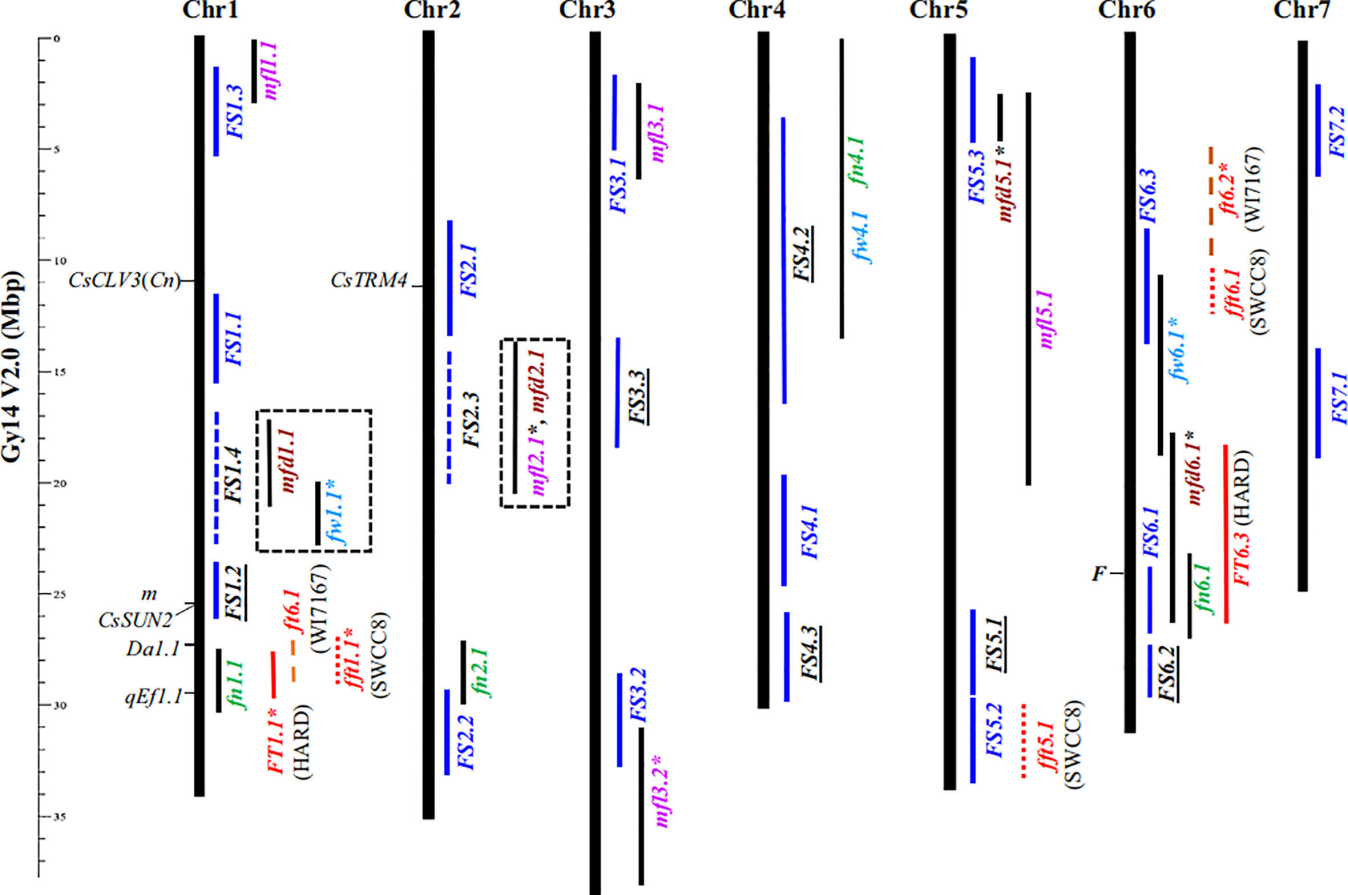
Physical locations of 21 consensus fruit size-related QTL (Sheng et al., 2019). Consensus fruit size QTL is indicated by blue vertical bars.

Before the entire cucumber genome was successfully sequenced, many researchers had studied the quantitative trait loci (QTLs) related to the shape and size of cucumber fruits, but almost no genes were cloned (Yuan et al., 2007; Yuan et al., 2008; Wang et al., 2009). At present, there are many reports on the genetic analysis, location, gene cloning and functional research of cucumber fruit’s shape, size, and length. Although many QTLs linked to the shape or size of cucumber fruit have been identified in recent years, very few of these QTLs have yet to be cloned. Qi et al. (2013) obtained five QTLs (*fl1.1, fl3.1, fl4.1, fl4.2* and *fl6.1*) related to cucumber fruit length by analyzing the resequencing results of 115 cucumber materials. Later, Wei et al. (2014) used the inbred lines CC3 and NC76 that differ greatly in fruit length to construct an F_2_ population, and then analyzed the length of commercial as well as mature fruit, which yielded eight QTLs relevant to fruit length. Weng et al. (2015) used different isolated populations to investigate fruits’ phenotype at different development stages, thereby obtaining 12 QTLs related to fruit size and shape. Zhu et al. (2016) found seven QTLs related to fruit length and eight QTLs related to fruit diameter by using a F_2_ population. Among the above loci, the QTL located by different researchers but which overlap in the cucumber chromosome position is referred to as the consensus QTL. Weng et al. (2015) put forward a model to explain the differences in cucumber fruit shape based on 12 consistent QTLs—*FS1.1*, *FS1.2*, *FS2.1*, *FS2.2*, *FS3.1*, *FS3.2*, *FS3.3*, *FS4.1*, *FS5.1*, *FS6.1*, *FS6.2*, *FS7.1*—among them, *FS1.1* plays a decisive role in fruit length, whereas only *FS3.3* regulates fruit elongation. Yet the model can only explain about 40% of the fruit length variation effect, which suggests other genes contribute to controlling fruit size. In later studies, Sheng et al. (2019) and Pan et al. (2020) added another nine consistent QTLs (*FS1.3*, *FS1.4*, *FS2.3*, *FS4.2*, *FS4.3*, *FS5.2*, *FS5.3*, *FS6.3*, *FS7.2*) to the cucumber fruit’s shape regulation model (**Figure 1**); among them, only *FS4.3* regulates the fruit length of cucumber. Most of these 21 QTLs can be detected in many different populations of cucumber plants, which also shows that their regulation can cover most of the extant trait variation in the shape of cucumber fruit.

Although researchers have performed much QTL mapping and analysis of cucumber fruit shape, many QTLs have not been cloned. With more in-depth basic research, increasingly more reports are becoming available on the fine mapping, gene cloning, and molecular mechanism of cucumber fruit concerning its size and shape. For example, Pan et al. (2017) used different populations to locate candidate genes responsible for round cucumber via marker linkage analysis, these being homologous to tomato *SlSUN* genes. Wu et al. (2018) located the candidate gene of *FS2.1* to be *CsTRM5*, which belongs to the homologous gene *SlTRM5*, a member of the tomato TONNEAU1 Recruiting Motif (TRM) family. In other work, by analyzing the sequence of 150 cucumber materials having different fruit lengths, Zhao et al. (2019) identified that the MADS-box family gene *CsFUL1* regulates the length of cucumber fruit. Through a QTL-seq analysis, Xu et al. (2020) located the major gene *CsFnl7.1* for cucumber neck length, which encodes a late embryogenesis abundant protein. More recently, using a NIL population, Che et al. (2023) identified the gene controlling the length of cucumber fruit as *CsRCR*, and analyzed the regulation mechanism of its alleles in the formation of cucumber fruit length.

By studying mutants of cucumber fruit length, researchers have also uncovered some quality trait genes that govern cucumber fruit length. For instance, Xin et al. (2019) obtained a short-fruit mutant *short fruit1*, generated by EMS (Ethylmethanesulfonate) mutation. Through BSA-seq and dCAPS marker screening, it was determined that *SF1* is the key gene regulating cucumber fruit elongation by regulating ethylene. Zhang et al. (2020) used *short fruit 2*, a natural mutant of short-fruit cucumber, to determine that the gene SF2 regulates cucumber fruit length by regulating cell proliferation, which encodes an HDC1 homolog. Cheng et al. (2021) recently mapped a candidate gene to *CsaV3_1G044310* by using a natural mutant *msf* (medium short-fruit), which encodes a homologous protein of the Arabidopsis type II inositol polyphosphate 5-phosphatase (type II 5 Ptase). Finally, Zhang et al. (2023) obtained a short-fruit mutant *sf4* by EMS mutation. Using map-based cloning, the gene that controls cucumber fruit elongation was identified as *Csa1G665390* on chr1, which encodes an O-linked N-acetylglucosamine (GlcNAc) transferase (OGT).

In this study, RIL (recombinant inbred line) populations were used to construct a genetic linkage map containing 600 bin markers by resequencing, and the population’s genetic background was analyzed. Based on the phenotypic data from spring 2021 and autumn 2022, QTL analysis of major loci of cucumber fruit length was carried out. Three coincident QTLs located on Chr2, Chr4, and Chr6 were obtained in both seasons, among which *Fl4.1* was the prominent interval, in that it contained 115 genes, of which 41 were significantly differentially expressed in the short-fruit line (S190) and long-fruit line (L67). Through gene function annotations we found that this interval contains a gene *Csa4G337340* encoding an auxin efflux carrier family protein; the expression level of this gene in the long-fruit lines is obviously down-regulated. According to the transcriptomics analysis, numerous genes were enriched in the plant hormone signal transduction pathway, and many differentially expressed transcription factors were found. Therefore, we speculate that plant hormones and their related genes in addition to a large number of transcription factors together play a pivotal role in regulating the length of cucumber fruit.

## Materials and methods

### Plants and growing conditions

The 151 RILs were obtained from a cross between ‘PI183967’ (*C. sativus* L. *hardwickii*) (HW) and a cultivated line, ‘XinTaiMiCi’ (XTMC). A fully randomized block design was used, in which three replicates were set in spring 2021 and likewise in autumn 2022, with five plants per replicate of one RIL line. The experimental materials were planted in the plastic arch shed at the experimental station of the Jimo Dipingxian Agricultural Cooperative of Qingdao Agricultural University, and subjected to conventional cultivation management practices in the field.

### Construction of high-density genetic map and background analysis of segregation population

The genetic background of the 151 RIL lines was analyzed by high-throughput sequencing, and a high-density genetic linkage map was built using the sequencing results.

### Phenotype data collection

We collected data on fruit length (‘Fl’, in cm; that is, the length from the apex of fruit to the pedicel attachment), measured according to the standards published by Yuan et al. (2008). Two fruits were measured per plant, and averaged over all plants to represent the fruit length of one RIL line.

### QTL analysis

All the genotype data from the RIL population plants were used to perform the linkage analysis using QTL IciMapping software (Meng L. et al., 2015).

### RNA extraction and quality testing

To reduce the differential genetic background as much as possible, we selected RIL-67 (L67) with the longest fruit length and RIL-190 (S190) with the shortest fruit length, for our investigation of the two seasons’ result and conducted the transcriptomics analysis on their commercial fruits. Selecting the fruit 15 days after flowering, remove the fruit stalk, fully grind the remaining fruit in a mortar filled with liquid nitrogen, and take 0.2g for the determination of RNA-seq. Three fruits were selected from each plant as three groups of biological repeats. Total RNA of each was isolated with an RNA extraction kit (Tiangen). The purity and concentration of each RNA sample were assessed with a NanoDrop 2000 spectrophotometer (Eppendorf, USA), using Agient2100/LabChip GX to accurately check the integrity of RNA, and detect the qualified RNA for library construction.

### Construction and quality control of cDNA library

The main process of cDNA library construction went as follows: (1) mRNA was isolated from total RNA using oligo-dT magnetic beads; (2) a fragmentation buffer was added to randomly interrupt mRNA; (3) mRNA was used as a template to synthesize cDNA, and the latter purified; (4) this purified double-stranded cDNA was repaired at the end, appended with poly-A and connected with a sequencing linker, after which the fragment size was selected by AMPure XP beads; (5) finally, the cDNA library was obtained by PCR enrichment.

After completing the library construction, the Qubit 3.0 fluorescence quantifier was used for preliminary quantifications, and the concentration should be > 1 ng/ μL. Next, the inserted fragments of the library were detected by the Qsep400 high-throughput analysis system. Finally, the effective concentration of a given library (effective concentration of the library > 2 nm) was accurately quantified via real-time PCR, to ensure the quality of each library.

### Sequencing and data analysis

All libraries were sequenced on the Illumina NovaSeq6000 platform at Biomarker Technologies (Qingdao). Sequencing data were analyzed by the bioinformatics analysis process provided by BMKCloud (www.biocloud.net).

### qRT-PCR verification

To verify the expressed genes in the transcriptomics data, eight differentially expressed genes (DEGs) were included for the qRT-PCR assay. Primer information for the qRT-PCR can be found in **Supplementary Table S1**, for which the Actin-F/R primers served as the control.

## Results

### Construction of a high-density genetic map and background analysis of the RILs

**Figure 2:**
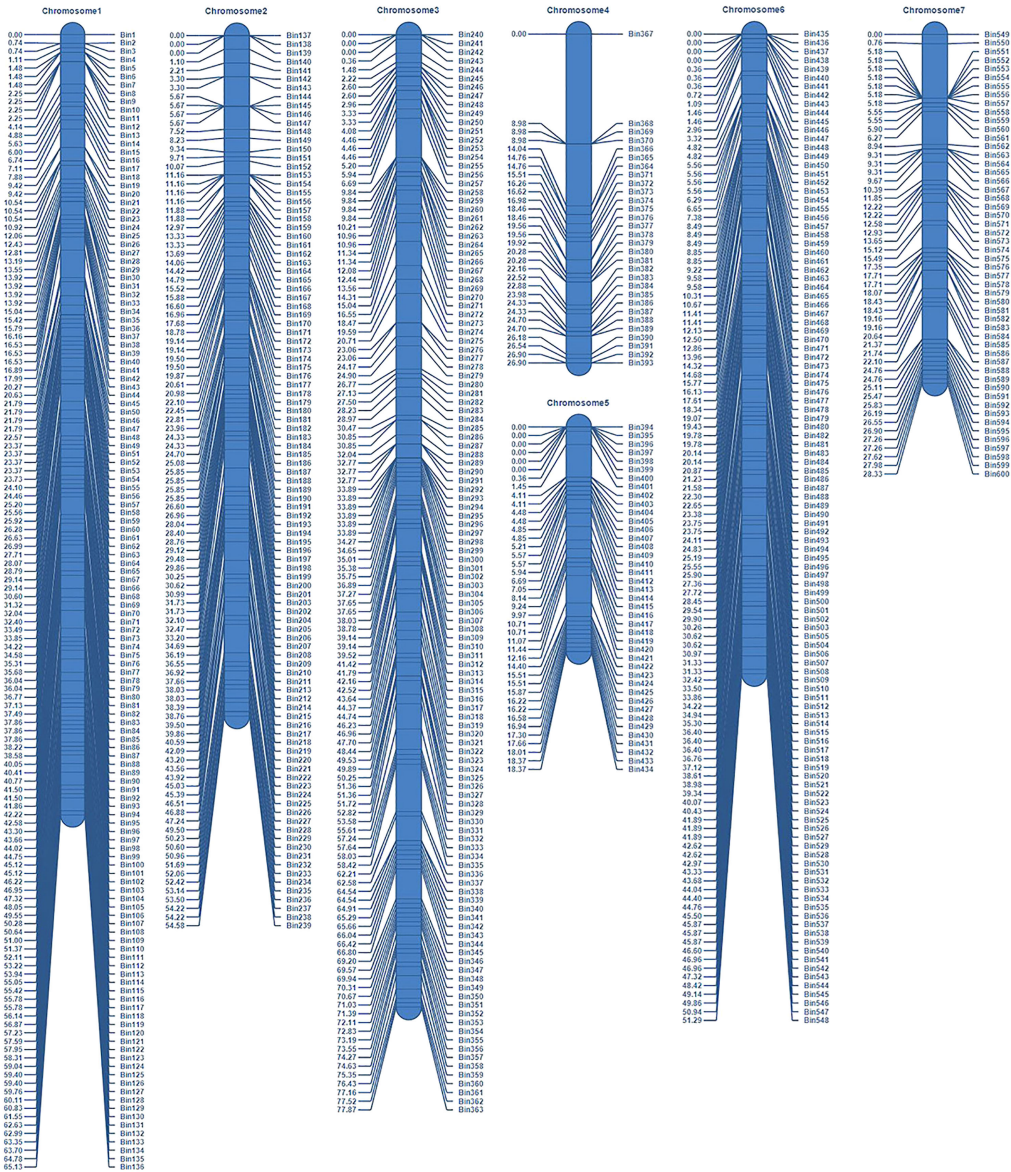
Schematic diagram of genetic map.

**Figure 3:**
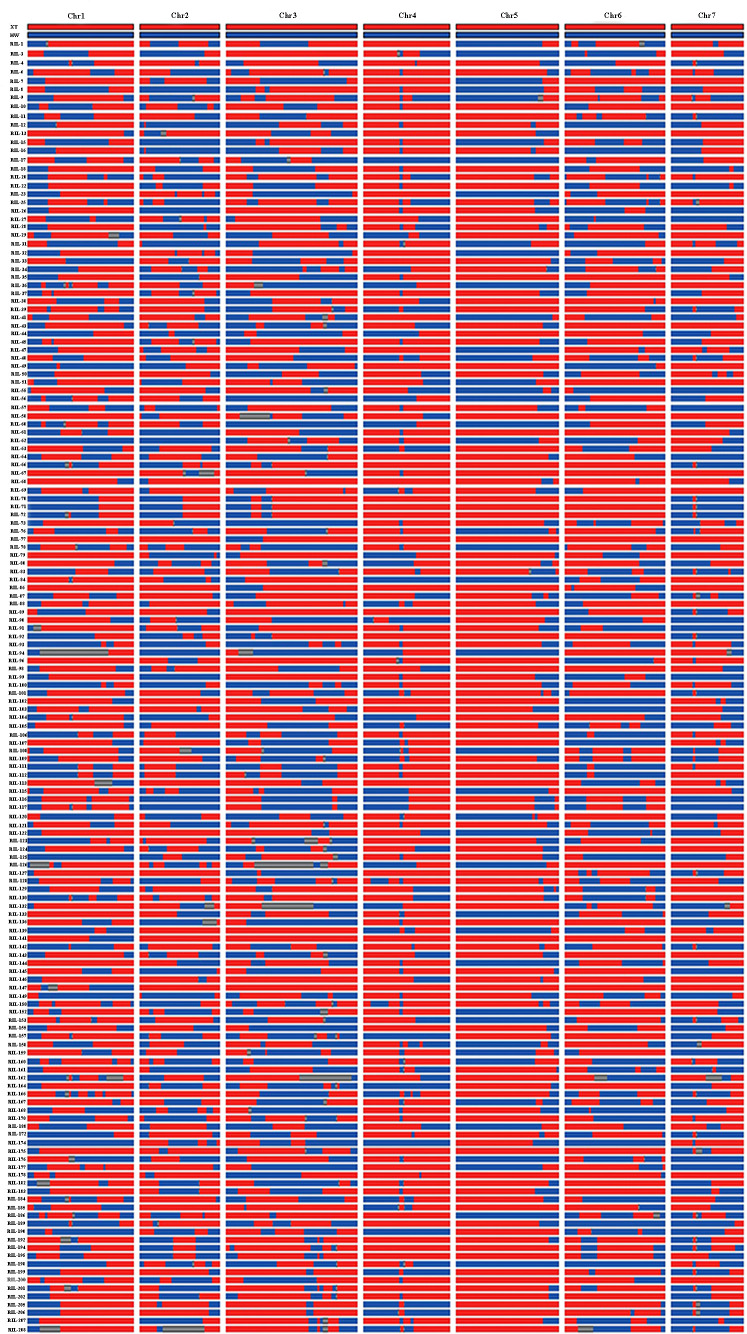
Detection results of genetic background of RIL population. Red stands for ‘Xintaimici’, blue stands for ‘Hardwickii’, gray stands for hybrid fragments.

The parents and 151 RIL lines were resequenced to construct a high-density genetic map (**Figure 2**). The total length of this map was 322.47 cM, which included seven linkage groups corresponding to cucumber chromosomes, with a total of 600 bin markers and an average minimum spacing of 0.45 cM (**Table 1**). By comparing the 151 RIL lines with the genomes of their parents, the genetic background and prospect of RIL populations were analyzed (**Figure 3**).

**Table 1:** Summary of genetic map information.

Linkage group	Chromosome length (cM)	Marker number	Average distance (cM)
Chr1	65.13	136	0.48
Chr2	54.58	103	0.53
Chr3	77.87	124	0.63
Chr4	26.90	30	0.90
Chr5	18.37	41	0.45
Chr6	51.29	114	0.45
Chr7	28.33	52	0.54

### Phenotypic analysis of fruit length

**Figure 4:**
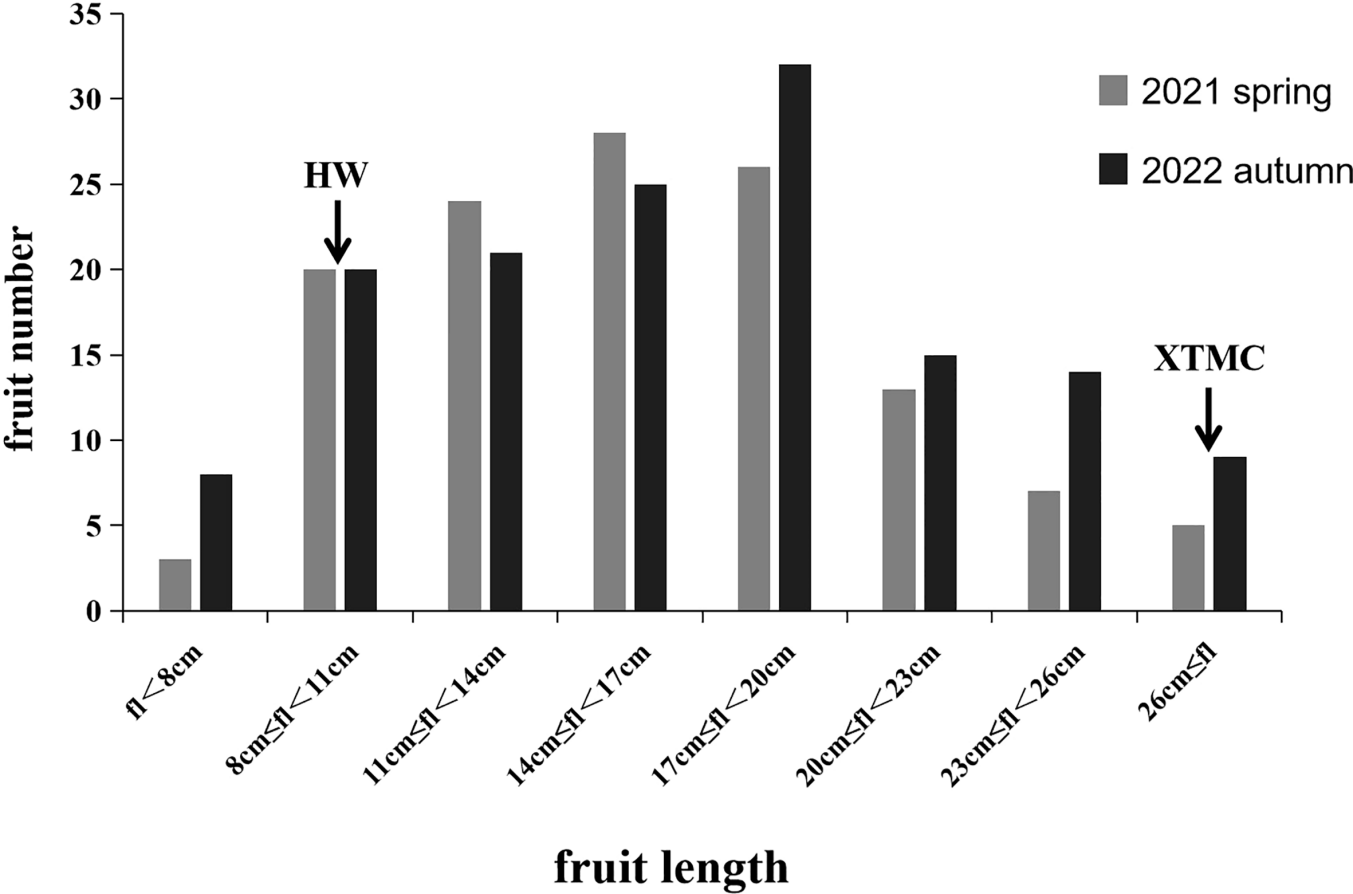
Statistical results of phenotypes of parents and RIL population. Gray is the investigation result in the spring of 2021, and black is the investigation result in the autumn of 2022. The two arrows respectively indicate the length range where the parent HW fruit and the parent XTMC fruit are located.

In the spring of 2021 and the autumn of 2022, the average length of HW fruit was 8.96 and 8.72 cm, respectively, while the corresponding average length of XTMC fruit was 30.78 and 30.12 cm. The fruit length of the RIL populations for the two seasons was mainly 6.75-29.6 cm and 6.89-30.86 cm, and it was continuously distributed among different lines (**Figure 4**). Among them, the fruit of RIL-190 was the shortest in the two seasons, at 6.75 and 6.89 cm, respectively, whereas that of RIL-67 was the longest, at 29.6 and 30.86 cm, respectively.

### QTL mapping results and gene analysis

**Figure 5:**
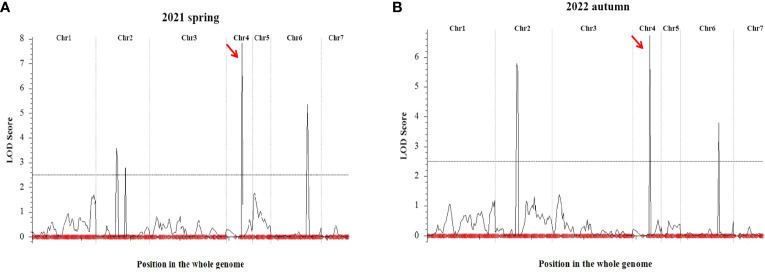
QTL mapping results of cucumber fruit length. QTL Mapping Results of Cucumber Fruit Length in 2021 spring **(A)** and 2022 autumn **(B)**. The symbols red arrow indicates the location of the major QTL.

**Figure 6:**
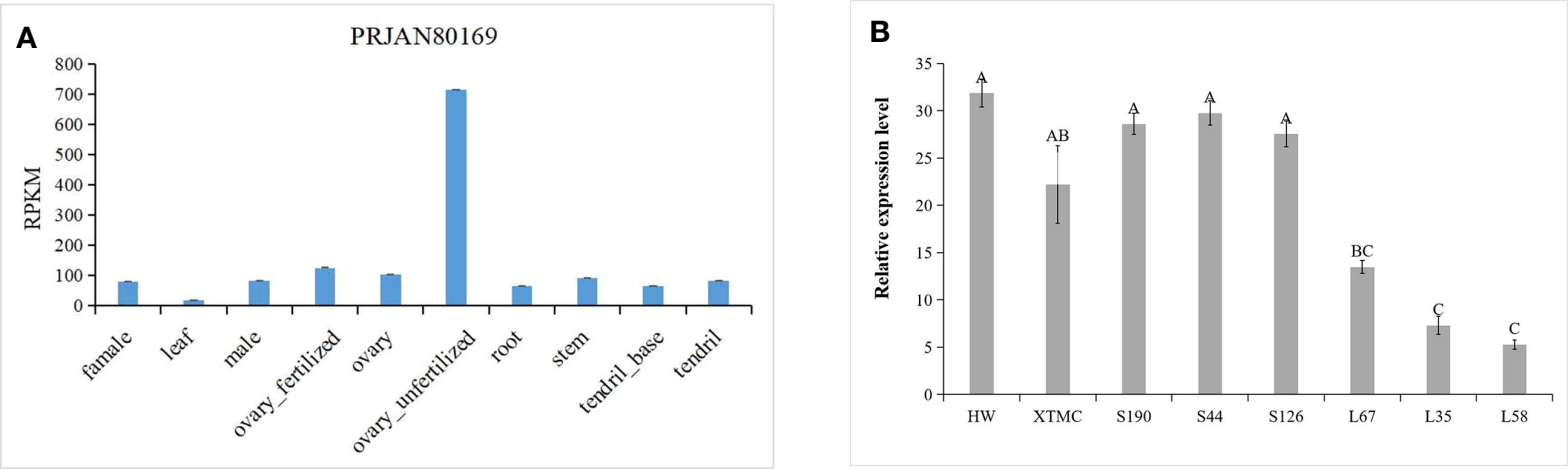
Expression of gene *Csa4G337340*. **(A)** Expression of *Csa4G337340* in different tissues of cucumber (Cucurbit Genomics Database); **(B)** Expression of *Csa4G337340* in parents and RIL lines with long and short fruits. Different capital letters in the figure indicate significant differences at the level of P < 0.05.

QTL IciMapping was used to analyze the fruit length of cucumber, with seven QTLs related to fruit length obtained. Among them, four QTLs (*Fl2.1*, *Fl2.2*, *Fl4.1*, and *Fl6.1*.) were obtained in spring 2021, the dominant locus being *Fl4.1*, and the LOD value was 7.82 (**Table 2**; **Figure 5A**); three QTLs (*Fl2.1*, *Fl4.1*, and *Fl6.1*.) were obtained in autumn 2022, with *Fl4.1* still the prominent locus, and the LOD value was 6.71 (**Table 2**; **Figure 5B**). In the mapping results of the two seasons, three QTL intervals on Chr2, Chr4, and Chr6 are coincident, indicating that the phenotype data of the RIL populations were relatively stable, with all major loci in the interval of 12862726–14137628 on Chr4. We identified 115 genes in the interval of *Fl4.1*, among which *Csa4G33734*0 was annotated as an auxin efflux carrier family protein; according to the cucumber genome database, this gene is expressed most in the unfertilized ovary (**Figure 6A**). We then selected two parents and three RIL lines with the longest fruit (L67, L35, and L58) and another three RIL lines with the shortest fruit (S190, S44, and S126), to determine this gene’s magnitude of expression. These results showed that the expression levels of *Csa4G33734*0 in long-fruit parent XTMC and long-fruit lines L67, L35, and L58 were significantly lower than those in short-fruit parent XTMC and short-fruit lines S190, S44, and S126 (**Figure 6B**).

**Table 2:** QTL intervals information statistics.

Seasons	QTLs	Chr	Position	Interval size (Kb)	LOD	PVE(%)	Add	Gene number
2021 spring	*Fl2.1*	2	11318282-11831887	513	3.58	7.138	-1.32	56
	*Fl2.2*	2	18154286-18620538	466	3.34	7.32	-1.34	56
	*Fl4.1*	4	12862726-14137628	1274	7.82	17.52	-2.46	115
	*Fl6.1*	6	24654259-24899860	245	5.34	10.96	-1.66	37
2022 autumn	*Fl2.1*	2	11318282-11831887	513	5.79	12.56	-1.96	56
	*Fl4.2*	4	12862726-14137628	1274	6.71	15.71	-2.60	115
	*Fl6.2*	6	24654259-24899860	245	3.78	8.04	-1.58	37

### Quality control of transcriptome sequencing data

After applying the quality control, a total of 42.99 Gb of data were obtained, and the percentage of Q30 bases in each sample was at least 95.85% (**Table 3**).

**Table 3:** Statistical results of sample data.

Samples	Clean reads	Clean bases	GC Content	%>Q30
S190-1	28,529,203	8,543,440,482	43.11%	96.75%
S190-2	25,316,164	7,579,461,884	42.84%	96.46%
S190-3	24,305,286	7,277,235,614	42.87%	96.35%
L67-1	20,946,293	6,271,925,220	43.07%	96.45%
L67-2	23,021,498	6,894,254,168	43.46%	96.15%
L67-3	21,461,558	6,427,601,460	43.22%	95.85%

In this study, cucumis _ sativus.chineselong _ v2.genome.fa served as the reference genome for the comparison of clean reads. HISAT2 software (Kim et al., 2015) was used to quickly and accurately compare clean reads with that reference genome, and thereby obtain their location information. Then, using StringTie (Pertea et al., 2015) to assemble the reads, we reconstructed the transcriptome for subsequent analysis. Employing the comparison rate, we sought to evaluate whether the selected reference genome assembly satisfied the information analysis needs. According to these results, the comparison rate between the reads of each sample and the reference genome was between 93.23% and 96.35% (**Table 4**). Hence, the reads checked via quality inspection were robust for use in further analysis.

**Table 4:** Sequence comparison results of sample sequencing data and reference genome.

Sample	Total Reads	Mapped Reads	Uniq Mapped Reads	Multiple Map Reads	Reads Map to ‘+’	Reads Map to ‘-’
L67-1	41,892,586	40,039,268 (95.58%)	39,240,965 (93.67%)	798,303 (1.91%)	20,516,556 (48.97%)	20,532,700 (49.01%)
L67-2	46,042,996	43,922,842 (95.40%)	42,531,472 (92.37%)	1,391,370 (3.02%)	22,924,808 (49.79%)	22,874,970 (49.68%)
L67-3	42,923,116	41,084,427 (95.72%)	40,279,820 (93.84%)	804,607 (1.87%)	21,041,465 (49.02%)	21,058,559 (49.06%)
S190-1	57,058,406	54,711,328 (95.89%)	53,526,474 (93.81%)	1,184,854 (2.08%)	28,116,643 (49.28%)	28,146,245 (49.33%)
S190-2	50,632,328	48,391,111 (95.57%)	47,463,663 (93.74%)	927,448 (1.83%)	24,779,636 (48.94%)	24,816,860 (49.01%)
S190-3	48,610,572	46,486,951 (95.63%)	45,601,998 (93.81%)	884,953 (1.82%)	23,790,786 (48.94%)	23,829,806 (49.02%)

(1)Sample: Sample analysis number;

(2)Total Reads: Clean Reads number, calculate by single end;

(3)Mapped Reads: Number of Reads mapped to the reference genome and its percentage in Clean Reads;

(4)Uniq Mapped Reads: The number of Reads mapped to the unique position of the reference genome and its percentage in Clean Reads;

(5)Multiple Map Reads: The number of Reads mapped to multiple locations in the reference genome and its percentage in Clean Reads;

(6)Reads Map to ‘+’: The number of Reads mapped to the positive strand of the reference genome and its percentage in Clean Reads;

(7)Reads Map to ‘-’: The number of Reads mapped to the negative strand of the reference genome and its percentage in Clean Reads.

**Figure 7:**
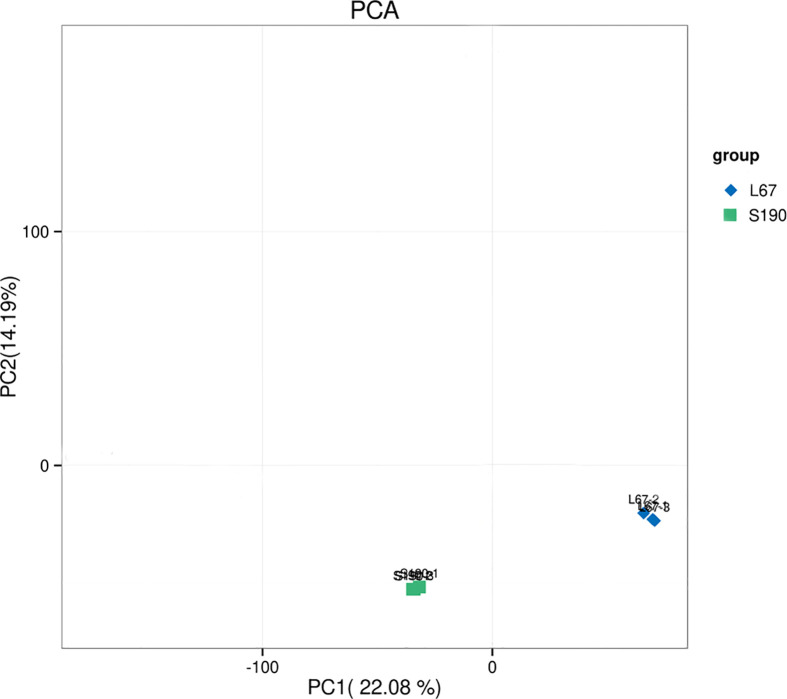
PCA analysis between different samples.

Using the DESeq package for R software, a principal component analysis (PCA) was performed using the expression levels of the samples. PCA analysis can cluster similar samples together, and the closer the distance among them, the higher the similarity between those samples. The PCA showed that the similarity between repeated samples is very high, confirming the samples’ highly reliability. However, evidently there is pronounced separation between certain differing samples, which indicated significant differences in gene expression among different samples fruits (**Figure 7**). Altogether, these results demonstrated that cucumber’s transcriptomic data can be credibly used for the subsequent analysis of DEGs.

### Differential expression genes analysis

A total of 9560 DEGs were obtained in S190 vs. L67, of which 4573 genes were up-regulated and 4987 genes were down-regulated. Several databases—CGO, GO, KEGG, KOG, NR, Pfam, Swiss-Pot, and eggNGO—were used to annotate the functions of DEGs. A total of 9314 genes were thus annotated, of which 7749 and 6399 genes were annotated in the GO (Gene Ontology) and KEGG (Kyoto Encyclopedia of Genes and Genomes) databases respectively (**Table 5**).

**Table 5:** Statistics of the number of DEGs.

DEG set	Total	CGO	GO	KEGG	KOG	NR	Pfam	Swiss-Pot	eggNGO
S190 vs L67	9314	3154	7749	6399	5039	9272	7679	6719	8757

**Figure 8:**
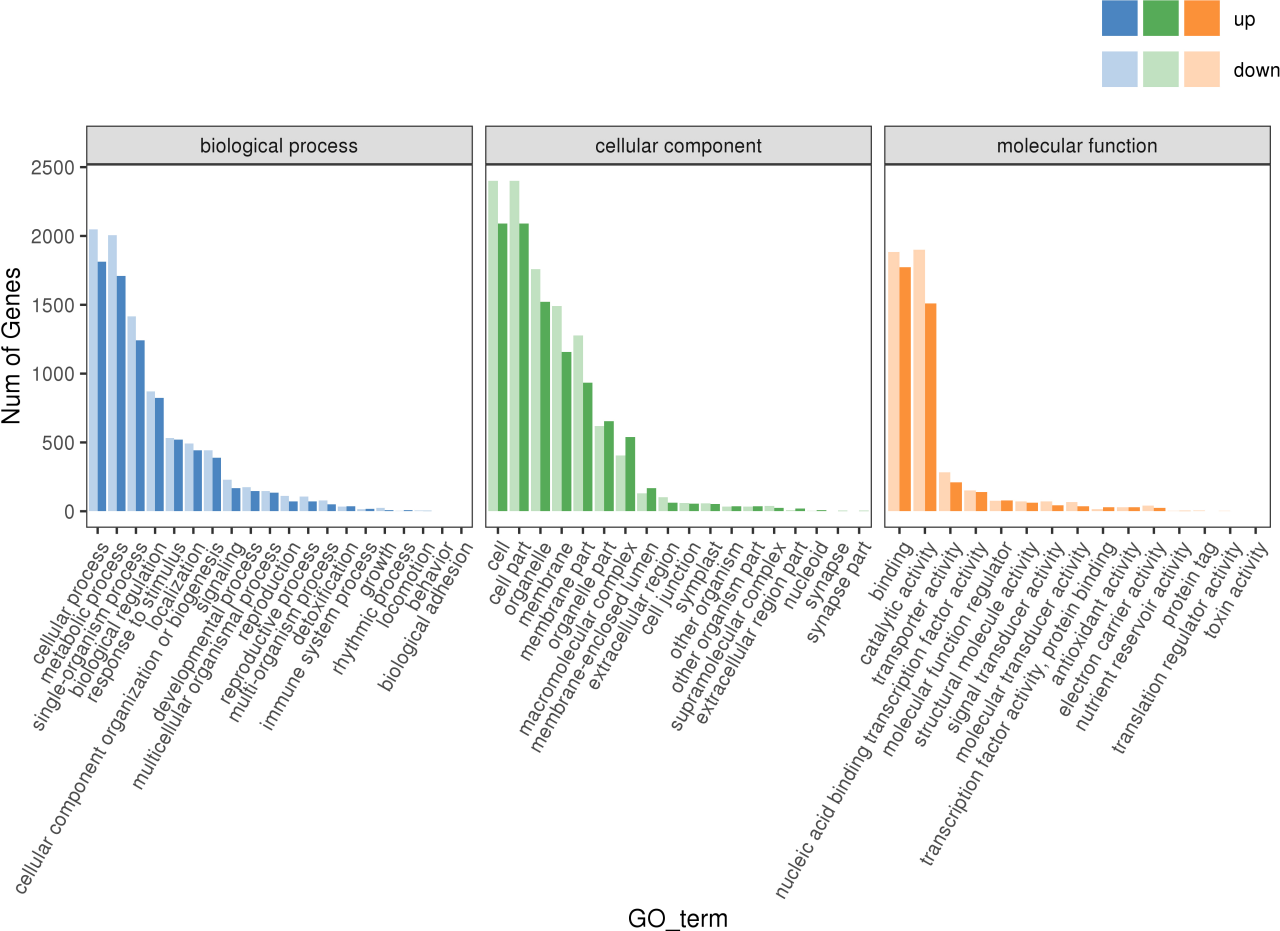
GO enrichment of DEGs. Blue histogram: the enrichment result of DEGs in biological process; Green histogram: the enrichment result of DEGs in cellular component; Qrange histogram: the enrichment result of DEGs in cellular component.

To further understand the functioning of these DEGs, their GO term enrichment analysis (P ≤ 0.05) was conducted. In the biological process (BP) group, the items with significant enrichment by DEGs mainly included ‘cellular process’, ‘metabolic process’, ‘single-organism process’, and ‘biological regulation’, among others (blue in **Figure 8**). In the cellular component (CC) group, the items with significant enrichment consisted mainly of ‘cell’, ‘cell part’, ‘organelle’, and ‘membrane’, and others (green in **Figure 8**). In the molecular function (MF) group, the items with significant enrichment chiefly comprised ‘binding’, ‘catalytic activity’, ‘transporter activity’, ‘molecular function regulator’, and so on (orange in **Figure 8**).

**Figure 9:**
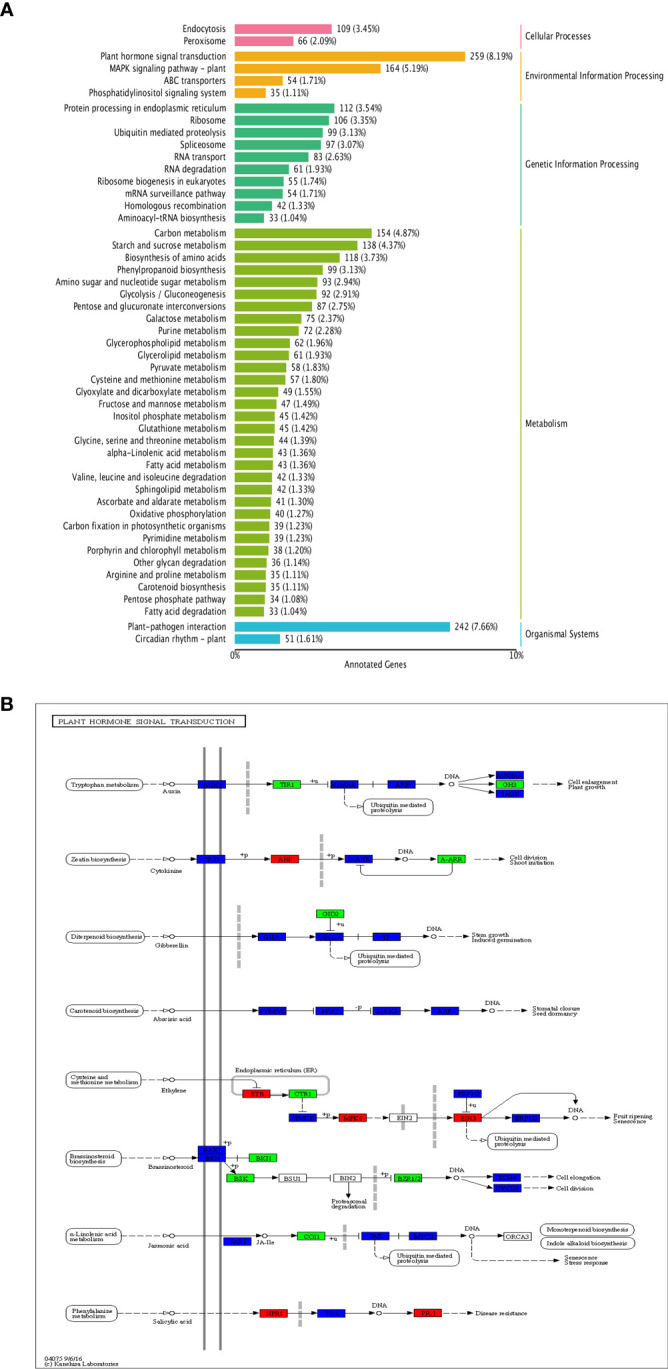
KEGG enrichment of DEGs. **(A)** KEGG enrichment of DEGs; **(B)** Distribution of DEGs in plant hormone signal transduction pathway.

KEGG enrichment analysis revealed the DEGs mainly enriched in several pathways, such as ‘plant hormone signal transduction’, ‘plant-pathogen interaction’, ‘MAPK signaling pathway-plant’, ‘carbon metabolism’ (**Figure 9A**); among them, the pathway having the most DEGs was ‘plant hormone signal transduction’, with a total of 259 genes enriched (**Figure 9B**). Overall, 67 DEGs were enriched in auxin signal transduction pathway, 25 up-regulated and 42 down-regulated; 27 DEGs were enriched in cytokinin signal transduction pathway, 9 up-regulated and 18 down-regulated. In addition, many DEGs were also enriched in the gibberellin, abscisic acid, ethylene, brassinolide, and salicylic acid signal transduction pathways (**Figure 9B**).

### Transcriptome level analysis of genes in the QTL intervals

**Figure 10:**
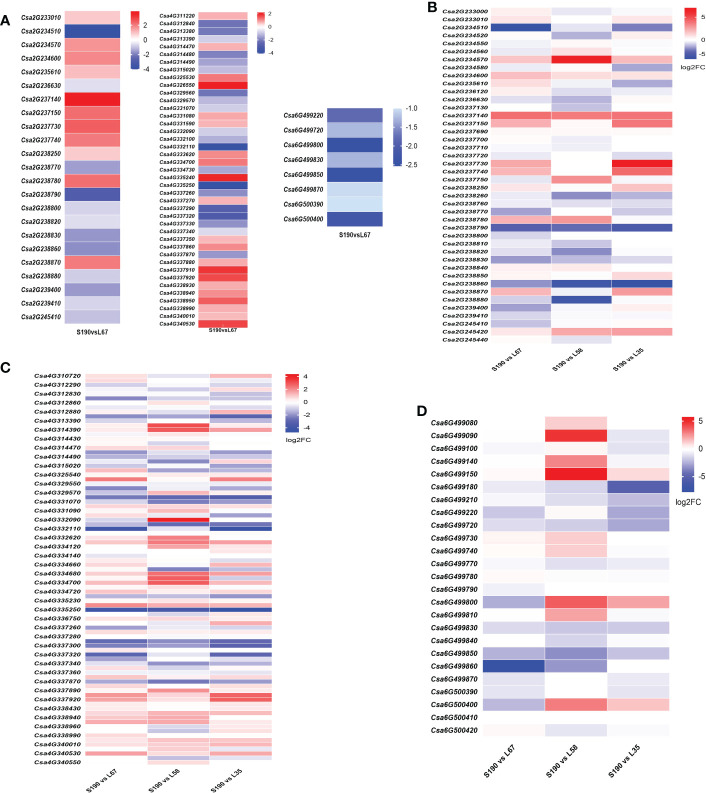
Gene expression in short fruit and different long fruit lines in the QTL intervals. **(A)** The expression of DEGs between RIL-190 and RIL-67 in the intervals of Fl2.1, Fl4.1 and Fl6.1; The expression of DEGs in the localization intervals Fl2.1 **(B)**, Fl4.1 **(C)** and Fl6.1 **(D)** in the short fruit line RIL-190 and the long fruit lines RIL-67, RIL-58 and RIL-35.

We also analyzed gene expression in the QTL intervals *Fl2.1, Fl4.1* and *Fl6.1*. Between S190 and L67, there were 23 DEGs in *Fl2.1*, of which 11 were up-regulated and 12 were down-regulated; likewise, 41 DEGs in *Fl4.1*, of which 21 were up-regulated and 20 were down-regulated; 8 DEGs in *Fl6.1*, all of which were down-regulated (**Figure 10A**). The expression of genes in the same three intervals was next analyzed between the shortest fruit line (S190) and the three longest fruit lines (L67, L35 and L58). In the range of *Fl2.1* there were seven genes up-regulated, such as *Csa2G234570*, *Csa2G234600*, and *Csa2G237140*, while ten genes were down-regulated, such as *Csa2G234510*, *Csa238790*, and *Csa2G238830*; (**Figure 10B**; **Supplementary Table S2**). In the range of *Fl4.1*, 27 genes (e.g., *Csa4G314390*, *Csa4G331080*, and *Csa4G334700*) were up-regulated while 25 other genes (e.g., *Csa4G314490*, *Csa4G335250*, and *Csa4G337340*) were instead down-regulated (**Figure 10C**; **Supplementary Table S2**). In the range of *Fl6.1*, only two genes *Csa6G499150 and Csa6G499730* were up-regulated in all three long-fruit lines, in contrast to seven genes, such as *Csa6G499180*, *Csa6G499770*, and *Csa6G499850*, that were down-regulated (**Figure 10D**; **Supplementary Table S2**). According to these gene expression patterns, we speculated that those genes up-regulated in long-fruit lines may participate in the positive regulation of the cucumber fruit length trait, while the down-regulated one participate in its negative regulation.

### Validation of RNA-Seq data by quantitative real time RT-PCR assays

**Figure 11:**
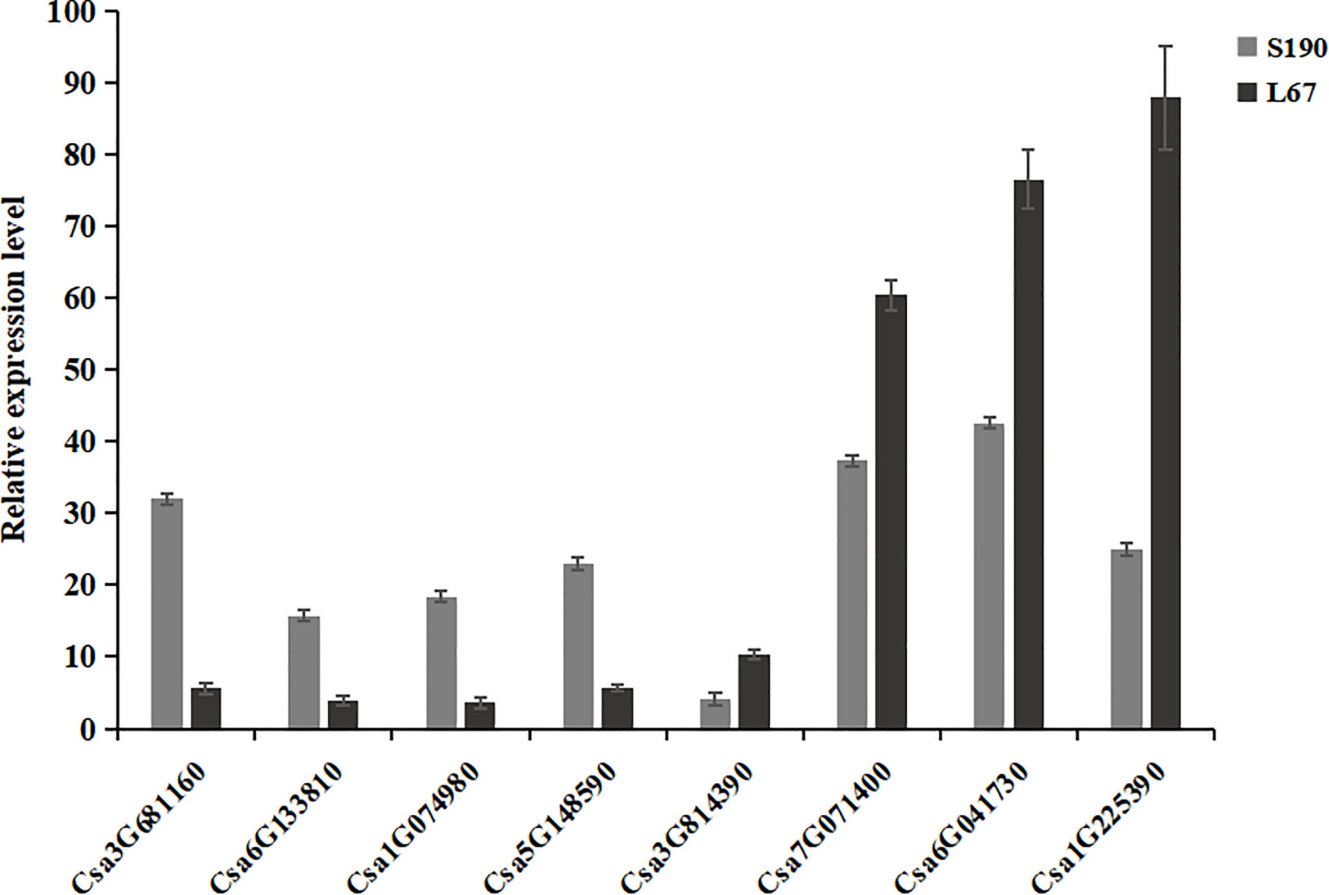
Verification of DEGs by qRT-PCR.

To verify the DEGs identified by RNA-Seq, eight DEGs were randomly selected and verified by qRT-PCR in the two sets of cucumber materials. Among those eight, four genes showed higher expression while the other four displayed lower expression in line S190. As **Figure 11** shows, for these genes, this expression pattern in the qRT-PCR assays was the same as that in the RNA-Seq data. Therefore, the RNA-Seq data were deemed highly reliable.

## Discussion

In this study, we analyzed the major QTLs controlling cucumber fruit length, and then explored the key genes and pathways related to cucumber fruit length by conducting a transcriptome analysis. The two-season phenotypic investigation demonstrated that the fruit length of 151 RIL lines was basically the same, irrespective of season (**Figure 4**), which suggests this trait is relatively stable in this RIL population. Among the QTLs distinguished in this study, the four QTLs of *Fl2.1*, *Fl2.2, Fl4.1*, and *Fl6.1* in spring 2021, and the three QTLs of *Fl2.1*, *Fl4.1*, and *Fl6.1* in autumn 2022, three were evident shared between seasons. Importantly, in both seasons the major QTL is *Fl4.1*, with LOD values of 7.82 and 6.71, respectively (**Figure 5**; **Table 2**). This locus is reportedly related to fruit length, fruit shape, and fruit stalk length, as shown in many previous studies. Indeed, has been identified as one of the consistent QTLs affecting cucumber fruit shape during fruit development in studies by Weng et al. (2015) and Sheng et al. (2019).

Auxin is a fundamental endogenous hormone that participates in many vital processes of plant growth and development, such as embryogenesis, organogenesis, cell determination and division, and tropic responses (Enders and Strader, 2015). These processes are finely coordinated by auxin, namely by requiring the polar distribution of auxin within tissues and cells. The intercellular directionality of auxin flow is closely related to the asymmetric subcellular location of PIN auxin efflux transporters (Zhou and Luo, 2018). In our study, further analysis of those genes in the interval *Fl4.1* shows that it contains 115 genes. According to their gene function annotations, the *Csa4G337340* gene in this region encodes an auxin efflux carrier family protein. According to this gene’s expression levels and patterning in different tissues of cucumber, as provided by the cucumber genome website, we find it maximally expressed in the unfertilized ovary (**Figure 6A**). From the RT-PCR, it is clear that expression levels of this gene are significantly lower in the long-fruit parent XTMC and the longest fruit RIL lines L67, L35, and L58 than those in the short-fruit parent HW and the shortest fruit RIL lines S190, S44-2, and S126 (**Figure 6B**). Combined with previous research findings, these results led us to speculate that *Csa4G337340* may affect the content of auxin in cucumber fruit by participating in its auxin transport, and thereby play a negative regulatory role in determining cucumber fruit length.

Plant endogenous hormones are crucial for normal fruit development, because they can individually or interactively influence plants’ tissue or organ development (Vogel, 2006; Su et al., 2011; Meng X. et al., 2015; Ma and Li, 2019). Notably, Zhang (2019) used the short fruit mutant *sf2* and wild-type cucumber to analyze the transcriptomics of their fruits at different developmental stages, finding that SF2 regulated the cell division of fruits through the synergistic regulation of various hormone pathways, such as IAA, GA, CK, ABA, JA and ETH, which then affected the fruit cell division. Recently, Hu et al. (2022) examined the hormones and hormone-related genes of the wild type and long-fruited mutant *lf*, showing that, compared with the wild type, the *lf* mutant’s fruit content of cytokinin and auxin changed significantly, as well as the expression of related genes; this indicates that auxin and cytokinin may be involved in the elongation of cucumber fruit cells. In the present study, from the transcriptomic analysis of fruits produced by shortest fruit line S190 and the longest fruit line L67 of the RIL populations, numerous DEGs were enriched in ‘plant hormone signal transduction’ pathway. Those enriched in auxin signal transduction pathway are the most abundant, totaling 67 DEGS, with 25 up-regulated and 42 down-regulated; another 27 DEGs were enriched in cytokinin signal transduction pathway (9 up-regulated, 18 down-regulated); In addition, a large number of genes are enriched in signal transduction pathways such as ethylene, gibberellin, abscisic acid, salicylic acid, and jasmonic acid. Therefore, we speculate that cucumber fruit length may be related to the content of hormones and the expression of hormone-related genes in fruit, a view consistent with other reported findings (Matsuo et al., 2012; Zhao et al., 2019).

**Figure 12:**
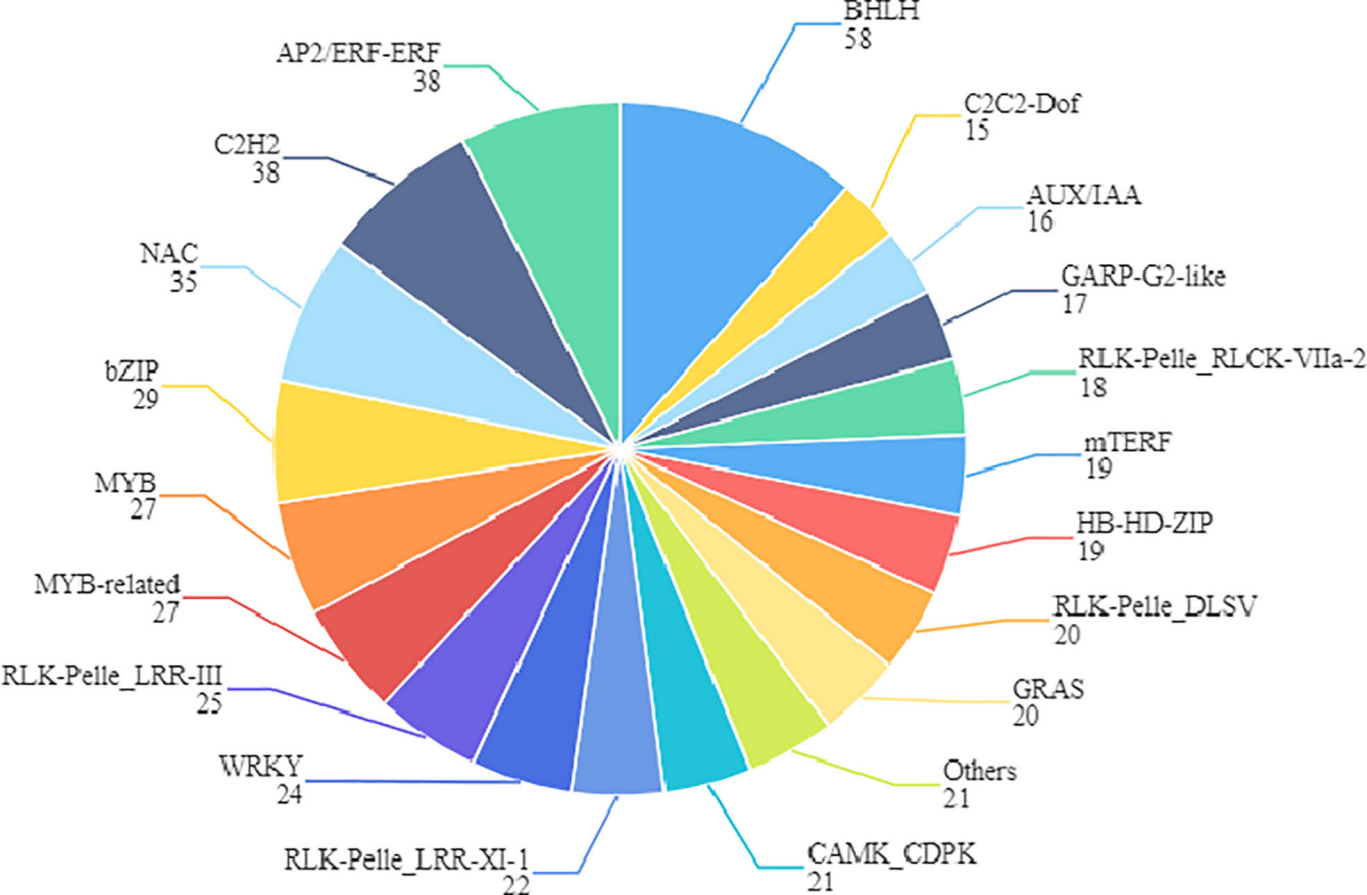
Family assignment of the 509 transcription factors. The name of transcription factor gene family and the number of genes it contains are marked in the figure.

Finally, we also found 509 differentially expressed transcription factors between S190 and L67, most of which belong to the BHLH, AP2/ERF-ERF, C2H2, NAC, Bzip, and MYB gene families. Many of them function in mediating cell division and expansion (**Figure 12**). In work by Jiang et al. (2015), a large number of transcription factors were found that could be related to cucumber fruit development, mainly concentrated in the MYB, BHLH, NAC, and ERF/AP2 gene families, including some reported transcription factors such as SPT, IND, CRC, FUL, SUP, and HAN (Heisler et al., 2001; Girin et al., 2011; Zhang et al., 2013). Hence, we speculate that transcription factors also figure prominently in regulating the determination of cucumber fruit length.

## Conclusion

A RIL population with 151 lines were derived from the cross between ‘HW’ and ‘XTMC’ and a high-density genetic map was constructed. Combined with the phenotypic data of different seasons, the QTL controlling the fruit length was located, and the major QTL was *Fl4.1*, which contained 115 genes. Based on the gene function annotations, we found a gene *Csa4G337340* encoding an auxin efflux carrier family protein, whose expression level in the long-fruit parent and three longest-fruit RIL lines is obviously down-regulated. Transcriptome sequencing was carried out on the fruits of shortest-fruit line S190 and longest-fruit line L67, and KEGG enrichment results showed that differentially expressed genes were significantly enriched in the plant hormone signal transduction pathway. In addition, 509 transcription factors were detected among the DEGs, these distributed in various transcription factor gene families, and many of them were reported to be involved in the regulation of phytohormone synthesis and metabolism. Accordingly, we conclude that the major gene controlling the fruit length of cucumber is located in the interval of *Fl4.1*, and the gene *Csa4G337340* in this interval may be involved in the negative regulation of fruit length. In addition, genes related to plant hormone signal transduction and several transcription factors are also involved in the regulation of cucumber fruit length.

## Data availability statement

The data presented in the study are deposited in the NCBI database repository, accession number PRJNA1005009.

## Author contributions

YX, YC and YM were mainly responsible for the investigation and statistics of phenotypic data. YX wrote the first draft and analyzed the transcriptomic data. FW and SX were responsible for the cultivation and management of cucumber materials. WZ conceived the idea and corrected the paper to present form. All authors read and approve the same for publication.
