# Supplementary material for: QTL mapping and transcriptomic analysis of fruit length in cucumber
Source: Front Plant Sci. 2023 Aug 21;14:1208675. doi: 10.3389/fpls.2023.1208675 (PMC10475832; doi:10.3389/fpls.2023.1208675)
Supplement: Supplementary file 1 [file Table_1.docx]

**Supplemental Table S1 qRT-PCR primer sequences**

| **NO.** | **Gene name** | **Forward primer（5’-3’）** | **Reverse primer（5’-3’）** |
| --- | --- | --- | --- |
| 1 | *Csa3G681160* | ATTCACCCTGTTTGCCCTGCTC | TATCCACACGACCAGTCCCTTAGC |
| 2 | *Csa6G133810* | ATTCACCCTGTTTGCCCTGCTC | TATCCACACGACCAGTCCCTTAGC |
| 3 | *Csa1G074980* | TGCAATGCCGATCTGTCTTCTTC | CCACGGGAGCTTTAGCATGAAAC |
| 4 | *Csa5G148590* | TCTTCCAATCAGACCAGGTTCGG | AGGTGCATCGTTACCTAATTCCG |
| 5 | *Csa3G814390* | TCGATTTCTCGCAATTGGAGGTTG | ACCTCTGAAGCCTGAACTCCAC |
| 6 | *Csa7G071400* | AGAAGAGCAAGATGCCCAAGTAGG | CTTCAGAACTGCACCCTTCTCC |
| 7 | *Csa6G041730* | TGTGCTGTAGTCTGCCTGTACC | TGTCATTGGCGAACTCCTGCTC |
| 8 | *Csa1G225390* | TGGCCGTTGGGATAGTTCATTCTC | AAGATTAACGTGTTCCTCCTTCCG |
| 9 | *CsActin* | ATTGTTCTCAGTGGTGGTTCTAC | CCTTTGAGATCCACATCTGCT |
