# Supplementary material for: QTL mapping and transcriptomic analysis of fruit length in cucumber
Source: Front Plant Sci. 2023 Aug 21;14:1208675. doi: 10.3389/fpls.2023.1208675 (PMC10475832; doi:10.3389/fpls.2023.1208675)
Supplement: Supplementary file 2 [file Table_2.docx]

**Supplementary Table S2** Functional annotations of genes that are up-regulated or down-regulated in long fruit lines in the range of *FL 2.1*, *FL 4.1* and *FL 6.1*.

| **NO.** | **Chromosome/QTL** | **Gene ID** | **Gene expression** | | | **Gene function annotation** |
| --- | --- | --- | --- | --- | --- | --- |
|  |  |  | **S190 vs L67** | **S190 vs L58** | **S190 vs L35** |  |
| 1 | Chr2/*FL2.1* | *Csa2G234570* | 1.66 | 6.69 | 1.91 | Pheromone receptor-like protein; contains IPR012442 (Protein of unknown function DUF1645) |
| 2 | Chr2/*FL2.1* | *Csa2G234600* | 1.83 | 1.07 | 0.90 | Peptidyl-prolyl cis-trans isomerase B; contains IPR002130 (Cyclophilin-like peptidyl-prolyl cis-trans isomerase domain), IPR011990 (Tetratricopeptide-like helical) |
| 3 | Chr2/*FL2.1* | *Csa2G237140* | 3.96 | 3.65 | 3.80 | Methylesterase |
| 4 | Chr2/*FL2.1* | *Csa2G237150* | 2.23 | 0.36 | 3.77 | COSII_At2g15890-like protein |
| 5 | Chr2/*FL2.1* | *Csa2G237690* | 0.40 | 0.35 | 0.32 | B3 domain-containing protein; contains IPR015300 (DNA-binding pseudobarrel domain) |
| 6 | Chr2/*FL2.1* | *Csa2G237730* | 2.47 | 0.04 | 7.11 | Unknown protein |
| 7 | Chr2/*FL2.1* | *Csa2G245420* | 0.76 | 2.48 | 2.66 | Phosphatidylinositol 4-kinase, putative; contains IPR015433 (Phosphatidylinositol Kinase), IPR016024 (Armadillo-type fold) |
| 8 | Chr2/*FL2.1* | *Csa2G234510* | -4.07 | -0.54 | -2.08 | Carboxylesterase 1; contains IPR013094 (Alpha/beta hydrolase fold-3) |
| 9 | Chr2/*FL2.1* | *Csa2G237710* | -0.26 | -0.36 | -0.09 | Serine/threonine protein kinase Smg1; contains IPR003152 (PIK-related kinase, FATC), IPR011009 (Protein kinase-like domain), IPR014009 (PIK-related kinase), IPR016024 (Armadillo-type fold) |
| 10 | Chr2/*FL2.1* | *Csa2G238260* | -0.42 | -1.85 | -1.27 | Long-chain-fatty-acid--CoA ligase; contains IPR000873 (AMP-dependent synthetase/ligase) |
| 11 | Chr2/*FL2.1* | *Csa2G238760* | -0.38 | -0.62 | -0.64 | Ring finger protein, putative; contains IPR013083 (Zinc finger, RING/FYVE/PHD-type) |
| 12 | Chr2/*FL2.1* | *Csa2G238790* | -2.85 | -2.45 | -3.19 | Desumoylating isopeptidase 2; contains IPR008580 (Domain of unknown function DUF862, eukaryotic) |
| 13 | Chr2/*FL2.1* | *Csa2G238820* | -0.61 | -1.88 | -0.17 | Pentatricopeptide repeat-containing protein, putative; contains IPR002885 (Pentatricopeptide repeat), IPR011990 (Tetratricopeptide-like helical) |
| 14 | Chr2/*FL2.1* | *Csa2G238830* | -1.65 | -1.16 | -0.71 | UPF0496 protein; contains IPR007749 (Protein of unknown function DUF677) |
| 15 | Chr2/*FL2.1* | *Csa2G238860* | -1.77 | -3.57 | -4.23 | At1g22630/F12K8_2; contains IPR001305 (Heat shock protein DnaJ, cysteine-rich domain) |
| 16 | Chr2/*FL2.1* | *Csa2G238880* | -0.84 | -3.59 | -0.20 | Non-symbiotic hemoglobin, putative; contains IPR000971 (Globin), IPR009050 (Globin-like) |
| 17 | Chr2/*FL2.1* | *Csa2G239410* | -0.75 | -0.21 | -0.24 | CASP-like protein; contains IPR006702 (Uncharacterised protein family UPF0497, trans-membrane plant) |
| 18 | Chr4/*FL4.1* | *Csa4G313890* | 0.37 | 3.09 | 0.42 | Citrate efflux MATE transporter |
| 19 | Chr4/*FL4.1* | *Csa4G314390* | 0.43 | 2.88 | 1.68 | Ethylene-responsive transcription factor 13; contains IPR016177 (DNA-binding, integrase-type) |
| 20 | Chr4/*FL4.1* | *Csa4G325540* | 0.29 | 0.42 | 0.45 | Regulatory-associated protein of mTOR; contains IPR004083 (Regulatory associated protein of TOR) |
| 21 | Chr4/*FL4.1* | *Csa4G326550* | 2.25 | 0.19 | 2.18 | Aminotransferase like protein; contains IPR005814 (Aminotransferase class-III), IPR015424 (Pyridoxal phosphate-dependent transferase) |
| 22 | Chr4/*FL4.1* | *Csa4G331080* | 0.76 | 0.74 | 0.60 | Saposin; contains IPR011001 (Saposin-like) |
| 23 | Chr4/*FL4.1* | *Csa4G332620* | 0.54 | 2.19 | 0.10 | MAC/Perforin domain containing protein; contains IPR020864 (Membrane attack complex component/perforin (MACPF) domain) |
| 24 | Chr4/*FL4.1* | *Csa4G333620* | 1.02 | 2.26 | 1.68 | Non-specific lipid-transfer protein; contains IPR000528 (Plant lipid transfer protein/Par allergen), IPR016140 (Bifunctional inhibitor/plant lipid transfer protein/seed storage helical domain) |
| 25 | Chr4/*FL4.1* | *Csa4G334120* | 0.07 | 1.58 | 0.48 | Receptor-like protein kinase; contains IPR011009 (Protein kinase-like domain) |
| 26 | Chr4/*FL4.1* | *Csa4G334680* | 0.87 | 2.47 | 1.67 | Unknown protein |
| 27 | Chr4/*FL4.1* | *Csa4G334700* | 1.16 | 2.81 | 1.20 | Thioredoxin; contains IPR005746 (Thioredoxin), IPR012336 (Thioredoxin-like fold) |
| 28 | Chr4/*FL4.1* | *Csa4G334720* | 1.03 | 0.37 | 0.74 | Ubiquitin-conjugating enzyme E2; contains IPR016135 (Ubiquitin-conjugating enzyme/RWD-like), IPR023313 (Ubiquitin-conjugating enzyme, active site) |
| 29 | Chr4/*FL4.1* | *Csa4G335230* | 0.20 | 0.53 | 0.29 | Transposon protein CACTA, En/Spm sub-class |
| 30 | Chr4/*FL4.1* | *Csa4G335240* | 1.99 | 1.40 | 1.30 | Acid phosphatase 1, putative; contains IPR005519 (Acid phosphatase (Class B)), IPR023214 (HAD-like domain) |
| 31 | Chr4/*FL4.1* | *Csa4G336750* | 0.56 | 0.72 | 0.40 | Receptor protein kinase-like protein; contains IPR001611 (Leucine-rich repeat), IPR003591 (Leucine-rich repeat, typical subtype), IPR011009 (Protein kinase-like domain), IPR013210 (Leucine-rich repeat-containing N-terminal, type 2), IPR013320 (Concanavalin A-like lectin/glucanase, subgroup), IPR025875 (Leucine rich repeat 4) |
| 32 | Chr4/*FL4.1* | *Csa4G337270* | 0.66 | 0.35 | 0.43 | KH domain-containing protein; contains IPR004087 (K Homology domain) |
| 33 | Chr4/*FL4.1* | *Csa4G337310* | 0.45 | 0.03 | 0.03 | RING finger protein; contains IPR013083 (Zinc finger, RING/FYVE/PHD-type) |
| 34 | Chr4/*FL4.1* | *Csa4G337860* | 1.02 | 0.34 | 0.69 | Singapore isolate B (sub-type 7) whole genome shotgun sequence assembly, scaffold_0; contains IPR015943 (WD40/YVTN repeat-like-containing domain) |
| 35 | Chr4/*FL4.1* | *Csa4G337880* | 0.73 | 0.04 | 0.59 | DNA helicase INO80; contains IPR000330 (SNF2-related), IPR001650 (Helicase, C-terminal), IPR020838 (DBINO domain), IPR027417 (P-loop containing nucleoside triphosphate hydrolase) |
| 36 | Chr4/*FL4.1* | *Csa4G337890* | 0.30 | 1.93 | 0.43 | Serine/threonine-protein kinase; contains IPR011009 (Protein kinase-like domain), IPR013320 (Concanavalin A-like lectin/glucanase, subgroup) |
| 37 | Chr4/*FL4.1* | *Csa4G337910* | 1.81 | 0.76 | 2.66 | L-threonine 3-dehydrogenase; contains IPR002085 (Alcohol dehydrogenase superfamily, zinc-type), IPR011032 (GroES-like), IPR013149 (Alcohol dehydrogenase, C-terminal), IPR016040 (NAD(P)-binding domain) |
| 38 | Chr4/*FL4.1* | *Csa4G337920* | 1.61 | 0.62 | 2.73 | Alcohol dehydrogenase-like protein; contains IPR002085 (Alcohol dehydrogenase superfamily, zinc-type), IPR011032 (GroES-like) |
| 39 | Chr4/*FL4.1* | *Csa4G338420* | 0.42 | 0.13 | 0.47 | General transcription factor IIH subunit; contains IPR005607 (BSD) |
| 40 | Chr4/*FL4.1* | *Csa4G338930* | 0.73 | 1.31 | 1.18 | T11I18.15 protein |
| 41 | Chr4/*FL4.1* | *Csa4G338950* | 1.42 | 1.49 | 0.46 | Hepatocyte growth factor-regulated tyrosine kinase substrate, putative; contains IPR008942 (ENTH/VHS) |
| 42 | Chr4/*FL4.1* | *Csa4G340000* | 0.52 | 1.05 | 1.02 | N-acetylglucosamine-1-phosphate transferase |
| 43 | Chr4/*FL4.1* | *Csa4G340010* | 0.61 | 0.85 | 1.25 | Phospho-N-acetylmuramoyl-pentapeptide-transferase; contains IPR000715 (Glycosyl transferase, family 4) |
| 44 | Chr4/*FL4.1* | *Csa4G340530* | 1.66 | 0.68 | 1.54 | GTP binding protein; contains IPR001806 (Small GTPase superfamily), IPR019341 (Alpha/gamma-adaptin-binding protein p34), IPR027417 (P-loop containing nucleoside triphosphate hydrolase) |
| 45 | Chr4/*FL4.1* | *Csa4G312290* | -0.95 | -0.24 | -0.86 | BAG family molecular chaperone regulator 4; contains IPR003103 (BAG domain), IPR019955 (Ubiquitin supergroup) |
| 46 | Chr4/*FL4.1* | *Csa4G312830* | -0.30 | -0.21 | -1.18 | Gamma-interferon-inducible lysosomal thiol reductase; contains IPR004911 (Gamma interferon inducible lysosomal thiol reductase GILT) |
| 47 | Chr4/*FL4.1* | *Csa4G312840* | -2.19 | -0.95 | -1.15 | Unknown protein |
| 48 | Chr4/*FL4.1* | *Csa4G312870* | -0.52 | -0.24 | -0.76 | Unknown protein |
| 49 | Chr4/*FL4.1* | *Csa4G313380* | -2.11 | -1.76 | -3.10 | Protein trichome birefringence-like 35; contains IPR025846 (PMR5 N-terminal domain), IPR026057 (PC-Esterase) |
| 50 | Chr4/*FL4.1* | *Csa4G314480* | -1.96 | -0.68 | -1.89 | Putative uncharacterized protein xy1420; contains IPR006342 (Methyltransferase FkbM), IPR013216 (Methyltransferase type 11) |
| 51 | Chr4/*FL4.1* | *Csa4G314490* | -1.48 | -0.99 | -1.09 | Unknown protein |
| 52 | Chr4/*FL4.1* | *Csa4G315020* | -1.09 | -0.24 | -1.80 | Wiskott-Aldrich syndrome protein family member 2; contains IPR003124 (WH2 domain) |
| 53 | Chr4/*FL4.1* | *Csa4G329550* | -0.39 | -0.40 | -0.46 | Alkylated DNA repair protein AlkB; contains IPR005123 (Oxoglutarate/iron-dependent dioxygenase), IPR027450 (Alpha-ketoglutarate-dependent dioxygenase AlkB-like) |
| 54 | Chr4/*FL4.1* | *Csa4G329560* | -2.22 | -0.43 | -1.67 | DNA binding protein, putative |
| 55 | Chr4/*FL4.1* | *Csa4G330070* | -2.47 | -2.90 | -3.32 | Unknown protein |
| 56 | Chr4/*FL4.1* | *Csa4G331070* | -0.85 | -2.35 | -1.87 | Myosin-13; contains IPR008545 (Protein of unknown function DUF827, plant) |
| 57 | Chr4/*FL4.1* | *Csa4G332100* | -1.32 | -2.80 | -2.57 | Ovate family protein 1; contains IPR006458 (Ovate protein family, C-terminal), IPR025830 (DNA-binding domain, ovate family-like) |
| 58 | Chr4/*FL4.1* | *Csa4G332110* | -4.05 | -0.62 | -4.78 | Serine/threonine-protein kinase 10; contains IPR011009 (Protein kinase-like domain) |
| 59 | Chr4/*FL4.1* | *Csa4G334730* | -1.43 | -1.27 | -1.96 | Putative receptor-like protein kinase; contains IPR001611 (Leucine-rich repeat), IPR011009 (Protein kinase-like domain), IPR013210 (Leucine-rich repeat-containing N-terminal, type 2) |
| 60 | Chr4/*FL4.1* | *Csa4G335250* | -4.13 | -3.77 | -4.69 | Pentatricopeptide repeat-containing protein; contains IPR002885 (Pentatricopeptide repeat), IPR011990 (Tetratricopeptide-like helical) |
| 61 | Chr4/*FL4.1* | *Csa4G337260* | -1.81 | -0.50 | -0.87 | Alphavirus core protein family; contains IPR021825 (Protein of unknown function DUF3411) |
| 62 | Chr4/*FL4.1* | *Csa4G337290* | -2.76 | -2.09 | -2.83 | AT3g08600/F17O14_7; contains IPR010605 (Protein of unknown function DUF1191) |
| 63 | Chr4/*FL4.1* | *Csa4G337300* | -2.35 | -1.84 | -3.10 | Unknown protein |
| 64 | Chr4/*FL4.1* | *Csa4G337320* | -3.00 | -0.62 | -2.95 | At2g30530/T6B20.12 |
| 65 | Chr4/*FL4.1* | *Csa4G337330* | -1.74 | -0.13 | -0.69 | E3 ubiquitin-protein ligase; contains IPR013083 (Zinc finger, RING/FYVE/PHD-type) |
| 66 | Chr4/*FL4.1* | *Csa4G337340* | -0.67 | -2.01 | -1.48 | Auxin efflux carrier family protein; contains IPR004776 (Auxin efflux carrier) |
| 67 | Chr4/*FL4.1* | *Csa4G337360* | -0.40 | -0.41 | -0.57 | Harpin-induced 1; contains IPR004864 (Late embryogenesis abundant protein, LEA-14) |
| 68 | Chr4/*FL4.1* | *Csa4G337870* | -1.66 | -2.46 | -1.86 | S-formylglutathione hydrolase; contains IPR000801 (Putative esterase) |
| 69 | Chr4/*FL4.1* | *Csa4G340540* | -0.13 | -1.23 | -0.63 | Calmodulin; contains IPR011992 (EF-hand-like domain) |
| 70 | Chr6/*FL6.1* | *Csa6G499150* | 0.25 | 5.83 | 0.89 | Unknown protein |
| 71 | Chr6/*FL6.1* | *Csa6G499730* | 0.41 | 1.21 | 0.08 | LMBR1 domain-containing protein-like protein; contains IPR006876 (LMBR1-like membrane protein) |
| 72 | Chr6/*FL6.1* | *Csa6G499180* | -0.82 | -1.44 | -5.22 | MATE efflux family protein; contains IPR002528 (Multi antimicrobial extrusion protein) |
| 73 | Chr6/*FL6.1* | *Csa6G499210* | -0.50 | -1.19 | -2.13 | Glucan 1,3-beta-glucosidase; contains IPR008999 (Actin cross-linking), IPR010431 (Fascin), IPR017853 (Glycoside hydrolase, superfamily) |
| 74 | Chr6/*FL6.1* | *Csa6G499720* | -1.26 | -1.62 | -2.77 | Homeobox-leucine zipper protein; contains IPR003106 (Leucine zipper, homeobox-associated), IPR009057 (Homeodomain-like) |
| 75 | Chr6/*FL6.1* | *Csa6G499770* | -0.47 | -1.10 | -0.76 | Ribosomal RNA small subunit methyltransferase F; contains IPR001678 (Bacterial Fmu (Sun)/eukaryotic nucleolar NOL1/Nop2p), IPR023267 (RNA (C5-cytosine) methyltransferase) |
| 76 | Chr6/*FL6.1* | *Csa6G499830* | -1.28 | -1.83 | -1.71 | Golgi apparatus membrane protein TVP23; contains IPR008564 (Protein of unknown function DUF846, eukaryotic) |
| 77 | Chr6/*FL6.1* | *Csa6G499850* | -2.44 | -3.42 | -1.95 | Putative hydrolase; contains IPR000073 (Alpha/beta hydrolase fold-1), IPR000639 (Epoxide hydrolase-like) |
| 78 | Chr6/*FL6.1* | *Csa6G500390* | -0.99 | -0.03 | -0.93 | Mitochondrial carrier protein, expressed; contains IPR002067 (Mitochondrial carrier protein), IPR023395 (Mitochondrial carrier domain) |
